# Therapeutic Potential of *Ocimum basilicum* in Diabetes–Malaria Comorbidity: Evidence From Parasitemia, Biochemical, and Coagulation Outcomes in Mice

**DOI:** 10.1155/jotm/6206620

**Published:** 2026-11-10

**Authors:** Opeyemi Oluwayemisi Ayodele, Olawunmi Rashidat Oyerinde, Oluwatosin Nancy Bello, Daniel Gbolahan Adeigbe, Gbenga Adegbite, Fehintoluwa Joy Femi-Olabisi

**Affiliations:** ^1^ Department of Biochemistry College of Basic and Applied Sciences Mountain Top University Prayer City Ogun State Nigeria mtu.edu.ng; ^2^ Department of Biological Sciences College of Basic and Applied Sciences Mountain Top University Prayer City Ogun State Nigeria mtu.edu.ng; ^3^ Department of Computer Science and Mathematics College of Basic and Applied Sciences Mountain Top University Prayer City Ogun State Nigeria mtu.edu.ng

**Keywords:** blood coagulation, cytokines, diabetes mellitus, malaria, *Ocimum basilicum*

## Abstract

Diabetes–malaria comorbidity presents a significant therapeutic challenge in endemic regions, as both conditions synergistically impair hepatic and renal function and lack targeted dual‐action treatments. Despite the reported antidiabetic and antiplasmodial properties of *Ocimum basilicum* (OCB), its efficacy in the concurrent management of these diseases remains unexplored. This study therefore evaluated the therapeutic potential of the hydromethanol extract of OCB on parasitemia, biochemical markers of organ function, inflammatory cytokines, and coagulation parameters in a murine model of diabetes–malaria comorbidity. Fifty‐six male mice were divided into eight groups (*n* = 7): control, diabetes only, malaria only, diabetes + malaria, malaria + OCB, diabetes + OCB, diabetes + malaria + OCB, and diabetes + malaria + metformin. Diabetes was induced by streptozotocin (40 mg/kg, i.p., for 5 consecutive days), followed by inoculation with *Plasmodium berghei*. Parasitemia was quantified by Giemsa‐stained thin blood films; fasting blood glucose (FBG), serum creatinine, urea, alanine aminotransferase (ALT), aspartate aminotransferase (AST), TNF‐*α*, interleukin‐6 (IL‐6), activated partial thromboplastin time, and prothrombin time were measured. The plant extract was profiled for phytochemicals using gas chromatography–mass spectrometry (GC–MS). OCB significantly reduced parasitemia relative to untreated infected controls (*p* < 0.05). In comorbid animals, OCB lowered FBG by 33.22%, creatinine by 70.58%, and urea by 26.32% compared with the untreated comorbid group. Hepatic enzyme activities (ALT and AST) were reduced more effectively by OCB than by metformin, indicating hepatoprotective activity. IL‐6 levels were also significantly suppressed (*p* < 0.01), while TNF‐*α* and coagulation parameters remained largely unchanged. The GC‐MS analysis identified forty‐three bioactive compounds, with predominant compounds including gamma‐sitosterol, 9,12‐octadecadienoic acid, *n*‐hexadecanoic acid, oleic acid, and N‐methyl‐1‐adamantane acetamide. These findings demonstrate that OCB exerts antiplasmodial, antihyperglycemic, and organ‐protective effects in diabetes–malaria comorbidity, highlighting its potential as a source of phytopharmaceutical agents for the integrated management of these co‐occurring diseases.

## 1. Introduction

Diabetes mellitus (DM) and malaria are major health challenges that increasingly intersect in populations where metabolic and infectious diseases coexist. Diabetes is characterized by persistent hyperglycemia resulting from impaired insulin secretion, insulin action, or both, and prolonged metabolic dysregulation can contribute to hepatic, renal, vascular, and other systemic complications [1]. Malaria, predominantly caused by *Plasmodium* species, remains an important cause of morbidity and mortality in tropical and subtropical regions, with sub‐Saharan Africa accounting for a substantial proportion of the global disease burden [2]. The coexistence of diabetes and malaria is of particular concern because each condition may adversely influence the course of the other. Diabetes‐associated metabolic dysfunction may compromise host responses to infection and increase the risk of adverse outcomes, while malaria‐associated inflammation, hemolysis, oxidative stress and organ injury may further disrupt glucose and metabolic homeostasis [3, 4].

The pathophysiological interaction between diabetes and malaria extends beyond hyperglycemia and parasitemia to involve oxidative stress, systemic inflammation, and tissue injury. Chronic hyperglycemia can promote oxidative and inflammatory disturbances that may influence the host response to *Plasmodium* infection. Conversely, malaria‐induced inflammatory responses, erythrocyte destruction, and hepatic stress may aggravate metabolic dysfunction and compromise organ function [3, 5]. These interactions create a therapeutic challenge because treatment of the individual diseases may not adequately address the broader metabolic, inflammatory, oxidative and hematological disturbances associated with their coexistence. Although conventional antidiabetic and antimalarial therapies remain central to disease management, concerns related to treatment‐associated adverse effects, accessibility, cost and the continuing emergence of antimalarial drug resistance have stimulated interest in complementary and alternative therapeutic strategies [6–8]. Consequently, agents with potential activity against multiple pathological processes associated with diabetes–malaria comorbidity warrant further investigation.

Plant‐derived products represent a potential source of such multitarget therapeutic agents because their diverse phytochemical constituents can act on several biological pathways simultaneously. *Ocimum basilicum* L. (OCB, sweet basil) is an aromatic medicinal plant widely used in traditional medicine and has attracted scientific interest because of its reported antioxidant, anti‐inflammatory, antimicrobial, and hypoglycemic activities. Its biological effects have been associated with phenolic compounds, flavonoids, and volatile constituents, including eugenol and linalool [9–11]. Experimental evidence therefore suggests that OCB may possess pharmacological properties relevant to some of the pathophysiological features of diabetes and infectious diseases. However, much of the available evidence has examined these activities in isolation, with studies generally focusing on either metabolic disorders, oxidative/inflammatory processes, or microbial infections rather than the integrated disease state produced by diabetes and malaria.

### 1.1. Research Gap and Novel Contribution

Despite the reported pharmacological activities of OCB, an important gap remains in understanding its therapeutic relevance under conditions in which diabetes and malaria occur concurrently. In particular, limited experimental evidence is available on whether OCB can simultaneously influence parasitemia and the metabolic and systemic disturbances associated with diabetes–malaria comorbidity. Furthermore, the effects of the plant extract on organ function biomarkers and hemostatic alterations in this combined disease model have received comparatively little attention. This is important because malaria can produce hematological and coagulation disturbances, while diabetes is also associated with altered hemostatic and vascular homeostasis. Evaluating these parameters together may therefore provide a more comprehensive assessment of therapeutic response than measuring glycemic or parasitological outcomes alone.

The present study addresses this gap by investigating the effects of a hydromethanol extract of OCB in BALB/c mice with streptozotocin‐induced diabetes and *Plasmodium berghei* infection. This integrated assessment provides insights into whether the extract can ameliorate multiple pathological consequences of diabetes–malaria comorbidity and establishes a basis for evaluating OCB as a potential multitarget phytotherapeutic candidate.

## 2. Methodology

### 2.1. Materials and Reagents

Streptozotocin (Santa Cruz, USA), metformin (Glucophage, Merck), methanol, phosphate‐buffered saline, trisodium citrate, sodium hydroxide, picric acid, and Giemsa stain were obtained from standard suppliers. Diagnostic assay kits for creatinine, urea, aspartate aminotransferase (AST), and alanine aminotransferase (ALT) were purchased from Randox Laboratories Ltd. Fasting blood glucose (FBG) was determined using Accu‐Chek and Fine Test glucometers with compatible strips. Other standard laboratory equipment included a UV–visible spectrophotometer, centrifuge, rotary evaporator, analytical balance, micropipettes, and glassware.

### 2.2. Study Design and Animal Handling

Fifty‐six (56) male BALB/C mice were procured from the Mountain Top University animal house (Ogun State, Nigeria). Animals were acclimatized for two weeks under standard housing conditions (12‐h light/dark cycle, room temperature, free access to food and water). All experimental procedures followed the Institutional Animal Ethics Committee (IAEC) guidelines. Institutional ethics approval was obtained with Reference No. CMUL/ACUREC/09/22/926.

The mice were randomly assigned to eight groups (*n* = 7):

1. Normal control
2. Diabetes only
3. Malaria only
4. Diabetes + malaria
5. Malaria + OCB extract
6. Diabetes + OCB extract
7. Diabetes + malaria + OCB extract
8. Diabetes + malaria + metformin

### 2.3. Collection and Extraction of Plant Material

Fresh OCB was collected in April 2023 from Ibafo, Ogun State, Nigeria, and authenticated by Dr. Nodza George (Department of Botany, University of Lagos). A voucher specimen (No. 10040) was deposited at the University herbarium. Leaves and roots were air‐dried at 40°C, pulverized, and stored in airtight containers at 4°C.

For extraction, 50 g of powdered leaves were macerated in 200 mL methanol and 200 mL distilled water (8:1°v/w) for 120 h with intermittent shaking. The filtrate was concentrated using a rotary evaporator and dried in a hot‐air oven at 50°C to yield a semisolid extract, which was stored at 4°C until use. A schematic illustration of the extraction process is presented in Figure 1.

**FIGURE 1:**
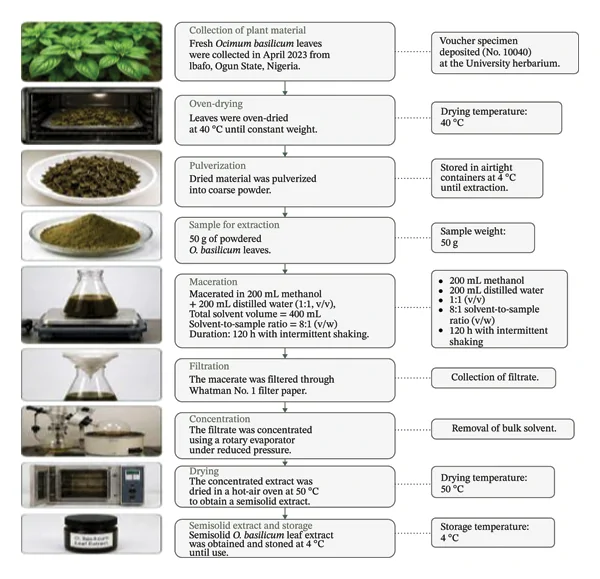
Schematic flowchart of the extraction procedure for *Ocimum basilicum* leaf extract.

### 2.4. Phytochemical Characterization by Gas Chromatography–Mass Spectrometry (GC–MS)

Phytochemical characterization of the plant extract was performed using GC–MS. The analysis was carried out using an Agilent 8860A gas chromatograph coupled to a 5977C inert mass spectrometer equipped with an electron‐impact (EI) ionization source (Agilent Technologies). Compound separation was achieved using an HP‐5 capillary column coated with 5% phenyl methyl siloxane (30 m × 0.32 mm internal diameter × 0.25 μm film thickness; Agilent Technologies).

Helium was used as the carrier gas at a constant flow rate of 1.573 mL/min, with an initial nominal pressure of 1.9514 psi and an average linear velocity of 46 cm/s. A 1 μL aliquot of the sample was injected in split mode at a split ratio of 50:1 and an injection temperature of 300°C. The purge flow was maintained at 21.5 mL/min for 0.50 min, with a total gas flow rate of 23.355 mL/min, while the gas saver mode was activated.

The oven temperature was initially set at 40°C and maintained for 1 min, followed by a temperature ramp of 10°C/min to 270°C, where it was held for 4 min. The total run time was 30.25 min, with a solvent delay of 3 min. The mass spectrometer was operated in electron‐impact ionization mode at 70 eV, with the ion source, quadrupole, and transfer line temperatures maintained at 230°C, 150°C, and 280°C, respectively. Mass spectra were acquired over a mass‐to‐charge ratio (m/*z*) range of 50–550 at a scan rate of 2.62 s/scan. The compounds detected were identified by comparing their measured mass spectral profiles with reference spectra in the NIST 14 Mass Spectral Library.

### 2.5. Induction of Diabetes and Malaria

Diabetes was induced by intraperitoneal administration of STZ (40 mg/kg) for five consecutive days following an overnight fast [12]. Hyperglycemia was confirmed on Day 14 in mice with FBG > 190 mg/dL. Malaria infection was induced with *P. berghei* (NK65 strain) obtained from the Institute of Advanced Medical Research and Training, University of Ibadan. On Day 9, diabetic groups were inoculated intraperitoneally with 0.2 mL of parasitized blood diluted in saline. Parasitemia was monitored by Giemsa‐stained thin blood smears from tail snips.

### 2.6. Treatment Protocol

OCB extract (100 mg/kg) and metformin (250 mg/kg) were administered orally for seven consecutive days. Normal control groups received PBS.

### 2.7. Sample Collection

On Day 25, animals were anesthetized and sacrificed by cervical dislocation. Blood was collected via ocular puncture into tubes with 3.2% trisodium citrate for coagulation assays. Plasma was separated by centrifugation (2500 g, 10 min). Liver and kidney tissues were excised, homogenized in PBS (1:8 w/v), and centrifuged (12,000 g, 10 min), and supernatants were stored at −20°C.

### 2.8. Biochemical and Immunological Assays

ALT and AST activities were determined using Randox kits based on 2,4‐dinitrophenylhydrazine methods. Serum creatinine and urea were quantified enzymatically using Randox kits, while total protein (TP) was determined by the Biuret method. Tumor necrosis factor‐alpha (TNF‐*α*) and interleukin‐6 (IL‐6) were assayed in the serum using enzyme‐linked immunosorbent assay (ELISA) kits obtained from Elabscience Biotechnology Inc., USA.

### 2.9. Hematological and Coagulation Parameters

Packed cell volume (PCV) was measured using the microhematocrit method. Prothrombin time (PT) and activated partial thromboplastin time (aPTT) were determined using standard coagulation kits in accordance with the manufacturer’s instructions.

### 2.10. Statistical Analysis

Data were expressed as mean ± SEM. One‐way ANOVA followed by Tukey’s post hoc test was performed using GraphPad Prism (v9.0.0.121). Significance was set at *p* < 0.05.

## 3. Results

### 3.1. GC–MS Identified Compounds in OCB Extract

The GC–MS analysis revealed a chemically diverse profile comprising 43 identified compounds, with fatty acids, phytosterols, flavonoid derivatives, terpenoid‐related compounds, hydrocarbons, phenolic compounds, and nitrogen‐ and sulfur‐containing compounds represented in the extract (Table 1). The major constituents based on peak area included *γ*‐sitosterol (6.83%), 9,12‐octadecadienoic acid (6.69%), *n*‐hexadecanoic acid (6.33%), 9‐octadecenoic acid (oleic acid; 5.30%), 3′,4′,5,6,7,8‐hexamethoxyflavone (4.01%), 3,5,6,7,8,3′,4′‐heptamethoxyflavone (3.88%), 9,17‐octadecadienal (3.54%), and N‐Methyl‐1‐adamantane acetamide (3.04%). The predominance of these compounds suggests that lipid‐derived constituents, phytosterols, and methoxylated flavonoids constitute important components of the extract.

**TABLE 1:** Phytocompounds identified in hydromethanol extract of *Ocimum basilicum L*.

Peak no	Retention time (min)	Area (%)	Name of compound	Molecular formula	Chemical group
1	7.195	6.33	n‐Hexadecanoic acid	C_16_H_32_O_2_	Fatty acid (saturated)
2	7.253	0.82	Scoparone	C_10_H_6_O_4_	Coumarin (benzopyrone)
3	8.145	6.69	9,12‐Octadecadienoic acid	C_18_H_32_O_2_	Fatty acid (polyunsaturated)
4	8.185	5.30	9‐Octadecenoic acid (Oleic acid)	C_18_H_34_O_2_	Fatty acid (monounsaturated)
5	8.231	1.14	Bicyclo[10.1.0]tridec‐1‐ene	C_13_H_22_	Unsaturated bicyclic hydrocarbon
6	8.283	2.01	5‐Eicosene	C_20_H_40_	Alkene hydrocarbon
7	9.667	1.91	Cyclohexane	C_6_H_12_	Cycloalkane hydrocarbon
8	10.125	1.42	2‐((Prop‐2 ‐ ynyloxy) carbonyl) benzoic acid	C_11_H_8_O_4_	Aromatic carboxylic acid/ester
9	10.228	1.37	1‐Hexadecanethiol	C_16_H_34_S	Alkanethiol
10	10.840	0.70	1‐Octen‐3‐yne	C_8_H_12_	Alkyne hydrocarbon
11	10.978	3.54	9,17‐Octadecadienal	C_18_H_32_O	Unsaturated aldehyde
12	11.069	1.29	Cyclopentene	C_5_H_8_	Cycloalkene hydrocarbon
13	11.315	1.24	5,9,13‐Pentadecatrien‐2‐one, 6,10, 14‐trimethyl (farnesyl acetone)	C_18_H_30_O	Sesquiterpenoid ketone
14	11.390	0.73	2 (1H)‐Naphthalenone, octahydro‐4a‐methyl‐7‐(1‐methylethyl)‐, (4a.alp ha.,7. beta.,8a. beta.)	C_14_H_24_O	Sesquiterpenoid ketone
15	11.584	2.45	3‐Methyl‐4‐(phenylthio)‐2‐prop‐2‐e nyl‐2,5‐dihydrothiophene 1,1‐dioxide	C_14_H_16_O_2_S_2_	Organosulfur heterocycle/sulfone
16	11.784	1.47	7,10‐Hexadecadienoic acid, methyl ester	C_18_H_32_O_2_	Fatty acid ester
17	11.859	1.12	Tricyclo[4.2.1.1 (2,5)]decane	C_10_H_16_	Tricyclic hydrocarbon
18	12.013	0.82	7‐Oxabicyclo[4.1.0]heptane, 1‐methyl‐4‐(2‐methyloxiranyl)	C_10_H_16_O_2_	Epoxide/cyclic ether
19	12.145	1.36	E‐3,13‐Octadecadien‐1‐ol	C_18_H_34_O	Unsaturated fatty alcohol
20	12.236	1.86	Bicyclo[2.2.1]heptan‐2‐ol, 2‐(1‐cyclopenten‐1‐yl)	C_11_H_16_O	Bicyclic alcohol
21	12.288	1.26	Tricyclo[4.2.1.1 (2,5)]decane	C_10_H_16_	Tricyclic hydrocarbon
22	12.374	3.39	Stigmasta‐3,5‐diene	C_30_H_50_	Steroid hydrocarbon (phytosteroid)
23	12.454	0.93	3,6,8,10‐pentaene‐8,9‐diamine	C_10_H_14_N_2_	Polyunsaturated diamine
24	12.528	2.02	Vitamin E	C_29_H_50_O_2_	Tocopherol
25	12.551	1.76	Hexahydropyridine, 4‐[4,5‐dimethoxyphenyl]	C_10_H_13_NO_2_	Piperidine derivative/alkaloid
26	12.671	1.50	4‐Dehydroxy‐N‐(4,5‐methylenedioxy‐2‐nitrobenzylidene)tyramine	C_17_H_16_N_2_O_5_	Schiff base/aromatic amine
27	12.723	1.33	4′,5,6,7,8‐Pentamethoxyflavone	C_18_H_16_O_7_	Flavonoid
28	12.900	3.21	Benzo[h]quinoline, 2,4‐dimethyl	C_15_H_13_N	Quinoline alkaloid
29	12.992	2.88	2‐Ethylacridine	C_13_H_11_N	Acridine alkaloid
30	13.209	6.83	Gamma‐sitosterol	C_29_H_50_O	Phytosterol
31	13.266/13.673	3.04	N‐Methyl‐1‐adamantane acetamide	C_13_H_21_NO	Adamantane carboxamide
32	13.369	3.88	3,5,6,7,8,3′,4′‐Heptamethoxyflavon	C_23_H_24_O_9_	Flavonoid
33	13.409	4.01	3′,4′,5,6,7,8‐Hexamethoxyflavone	C_23_H_24_O_9_	Flavonoid
34	13.472	2.82	1‐(3‐Chlorophenyl)‐3‐methyl‐1H‐pyrazol‐5‐amine	C_10_H_9_ClN_4_	Pyrazole amine
35	13.530	1.94	2′‐Hydroxypropiophenone	C_10_H_10_O_2_	Hydroxy aromatic ketone
36	13.604	2.10	1,2‐Bis(trimethylsilyl)benzene	C_16_H_28_Si_2_	Organosilicon aromatic hydrocarbon
37	13.690	1.78	Arsenous acid, tris(trimethylsilyl) ester	C_9_H_27_AsO_3_Si_3_	Organosilicon arsenic ester
38	13.987	2.39	1,2,5‐Oxadiazol‐3‐amine, 4‐(3‐methoxyphenoxy)‐	C_12_H_12_N_4_O_3_	Oxadiazole amine/aromatic ether
39	14.302	2.44	2‐Ethylacridine	C_14_H_11_N	Acridine alkaloid
40	15.000	2.88	2‐(Pyridin‐2 ylformamido) acetic acid	C_10_H_9_N_3_O_3_	Heteroaromatic amide/carboxylic acid
41	15.389	1.13	4‐tert‐Octylphenol	C_14_H_22_O	Alkylphenol
42	15.572	1.17	2′‐Hydroxy‐5′‐methylacetophenone	C_10_H_12_O_2_	Hydroxy acetophenone
43	16.019	1.75	4‐(4‐Hydroxyphenyl)‐4‐methyl‐2‐pentanone	C_14_H_18_O_2_	Hydroxy aromatic ketone

### 3.2. Effects of OCB on FBG

The effects of OCB extract on FBG are presented in Figure 2. Diabetic mice exhibited a marked elevation in FBG on Days 15 and 25 compared with the normal control, confirming persistent hyperglycemia in the diabetic groups. Treatment with OCB produced a clear reduction in FBG in the diabetic (Db + OCB), malaria–diabetic (Mal + Db + OCB), and malaria‐only (Mal + OCB) groups relative to their respective untreated counterparts. The reduction was evident across the disease conditions, indicating that OCB treatment was associated with improved glycemic control in both diabetes and diabetes occurring in combination with malaria. The response observed in the comorbid group is particularly relevant to the study objective, as it demonstrates that OCB retained its glucose‐lowering effect in the presence of *P. berghei* infection.

**FIGURE 2:**
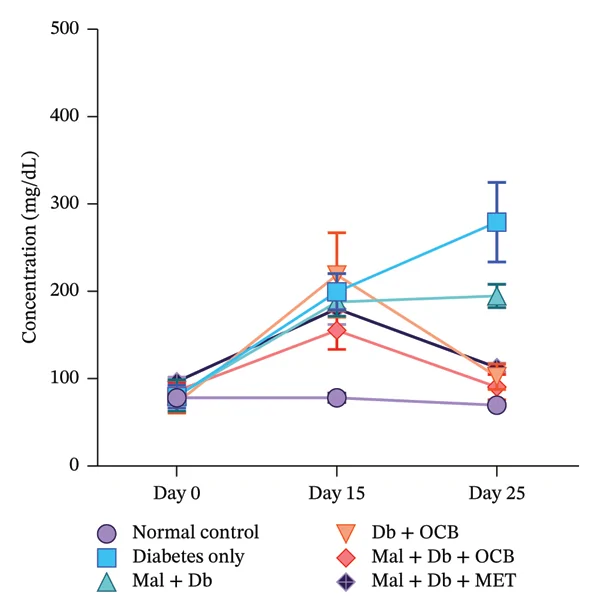
Effect of OCB on FBG levels in experimental mice.

MAL: malaria, Db: diabetic, OCB: *Ocimum basilicum*, MET: metformin.

### 3.3. Effects of OCB on Parasitemia

Figure 3 shows the effect of OCB treatment on parasitemia over the experimental period. Parasitemia remained highest in the malaria‐only group, indicating a substantial parasite burden in the absence of OCB treatment. In contrast, mice treated with OCB, particularly the Mal + OCB and Mal + Db + OCB groups, exhibited a lower parasitemia burden, with the treatment‐associated reduction becoming more apparent from Day 9 onwards. The persistence of this difference during the later phase of infection suggests that OCB treatment was associated with suppression of parasite proliferation or reduction in parasite burden. Importantly, the reduction was also observed in malaria + diabetic mice, indicating that diabetes did not completely abolish the antiparasitic response associated with OCB. Metformin also reduced parasitemia in the Mal + Db group at Day 25, although the pattern differed from that observed with OCB treatment.

**FIGURE 3:**
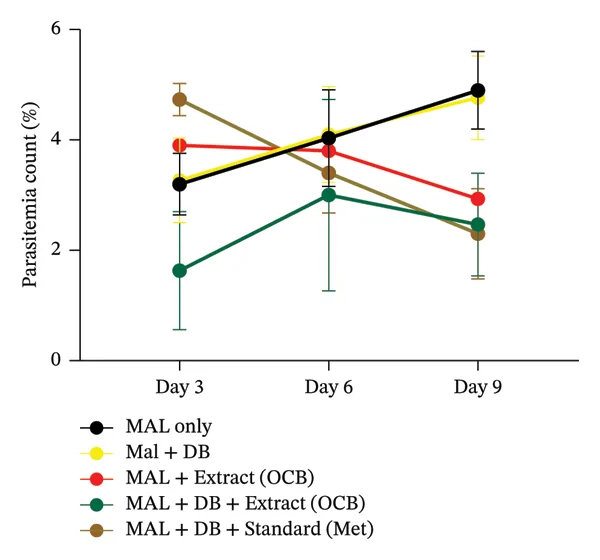
Effect of OCB on parasitemia count in experimental mice. NC: normal control, MAL: malaria, DB: diabetic, OCB: *Ocimum basilicum*, Met: metformin.

### 3.4. Effects of OCB on Body Weight

Infection with *P. berghei* and/or diabetes resulted in a progressive decline in body weight compared with the normal control, demonstrating the adverse systemic effects associated with the individual and combined disease conditions. OCB treatment attenuated this decline, with treated animals generally maintaining higher body weights than their corresponding untreated controls. By Day 25, preservation of body weight was particularly evident in the Db + OCB and Mal + Db + OCB groups (Figure 4). The greater maintenance of body weight in the comorbid treatment group suggests that OCB may have mitigated some of the metabolic and systemic consequences associated with the simultaneous presence of diabetes and malaria.

**FIGURE 4:**
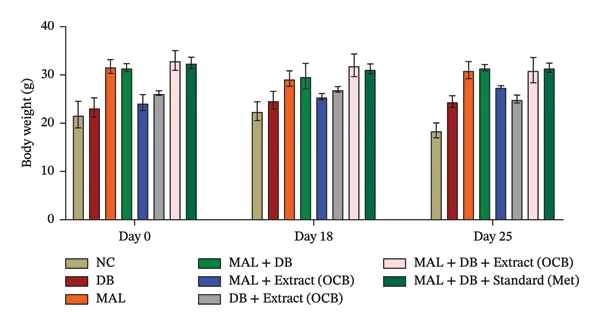
Effect of OCB on the body weight of experimental mice. NC: normal control, MAL: malaria, DB: diabetic, OCB: *Ocimum basilicum*, Met: metformin.

### 3.5. Effects on Hepatic Enzyme Activities

The effects of OCB on ALT and AST activities are shown in Figures 5 and 6, respectively. ALT activity was significantly elevated in the untreated diabetic, malaria, and malaria–diabetic groups compared with the normal control, indicating altered hepatic biochemical status under each disease condition. Treatment with OCB attenuated these elevations, suggesting an improvement in the altered ALT response associated with diabetes and/or malaria.

**FIGURE 5:**
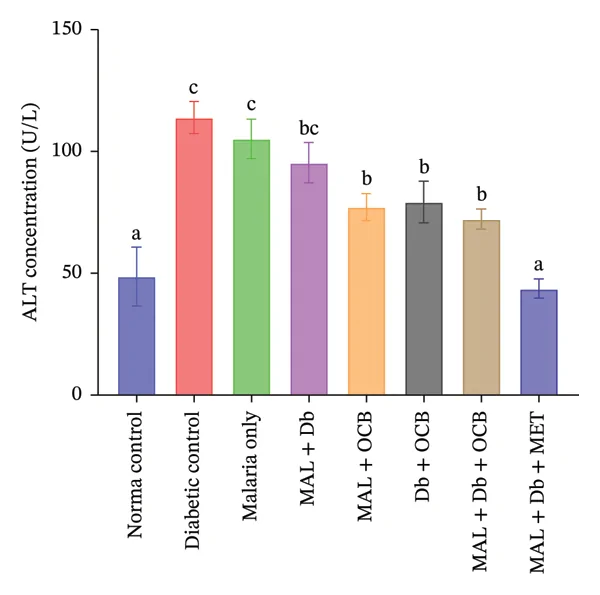
Effects of OCB on ALT concentrations in experimental mice. Data are presented as mean ± SEM; *n* = 7. Different letters on bars indicate a significant difference (*p* < 0.05). MAL: malaria, Db: diabetic, OCB: *Ocimum basilicum*, MET: metformin.

**FIGURE 6:**
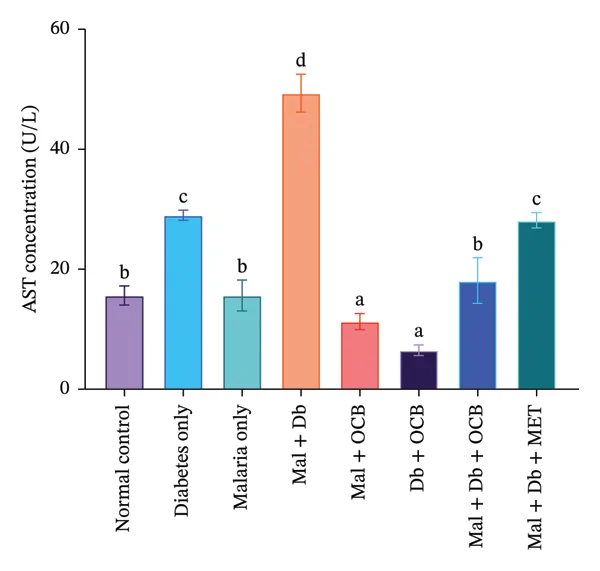
Effects of OCB on AST concentrations in experimental mice. Data are presented as mean ± SEM; *n* = 7. Different letters on bars indicate a significant difference (*p* < 0.05). Mal: malaria, Db: diabetic, OCB: *Ocimum basilicum*, MET: metformin.

A similar pattern was observed for AST, although the response varied according to disease condition. AST activity was significantly lower in the Db + OCB group than in the untreated diabetic group. In contrast, the Mal + Db group exhibited higher AST activity than both the malaria‐only and diabetes‐only groups, indicating a more pronounced alteration when the two conditions occurred concurrently. OCB treatment reduced AST activity relative to the untreated malaria groups. Taken together, the ALT and AST findings demonstrate that the comorbid condition was associated with greater disruption of hepatic enzyme profiles and that OCB treatment was accompanied by attenuation of these changes.

### 3.6. Effects on Renal Function Markers (Urea and Creatinine)

Urea concentration was elevated in the malaria‐only group, whereas treatment with OCB resulted in a significant reduction (*p* < 0.05) in the Mal + OCB group (Figure 7). This finding indicates that the malaria‐associated alteration in urea concentration was attenuated following OCB administration.

**FIGURE 7:**
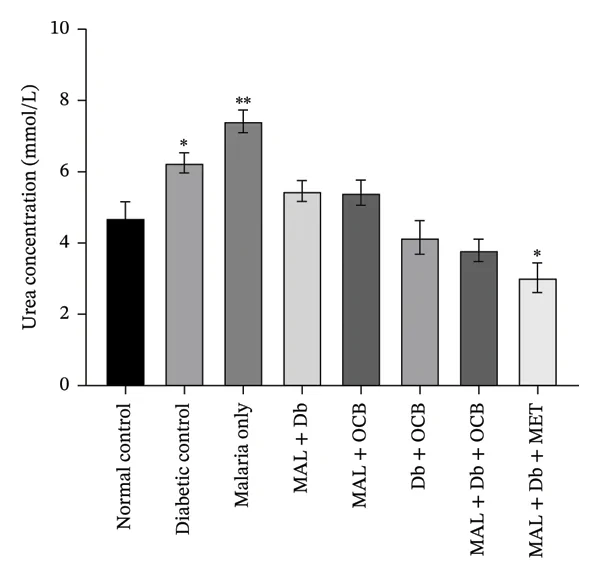
Effect of OCB on urea concentrations in mice. Data are presented as mean ± SEM; *n* = 7; ^∗^ *p* < 0.05, ^∗^^∗^ *p* < 0.01 compared to corresponding OCB‐treated groups. Mal: malaria, Db: diabetic, OCB: *Ocimum basilicum*, MET: metformin.

Creatinine concentrations were significantly lower in the Mal + OCB, Db + OCB, and Mal + Db + OCB groups than in their corresponding untreated groups. The treatment groups also showed significantly lower creatinine concentrations than the normal control (Figure 8). Thus, OCB administration was associated with a consistent reduction in circulating creatinine across the malaria, diabetes, and comorbid conditions. Because the direction and magnitude of renal markers were not identical for urea and creatinine, the findings suggest that the effects of disease and treatment on these two markers were not completely parallel.

**FIGURE 8:**
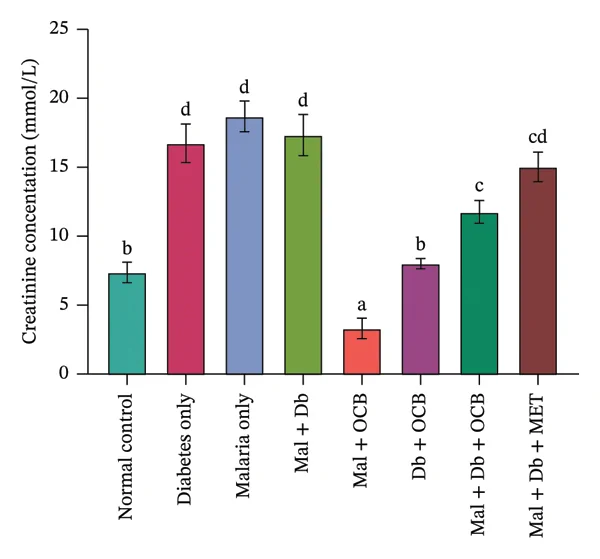
Effect of OCB on creatinine concentrations in mice. Data are presented as mean ± SEM; *n* = 7. Different letters on bars indicate a significant difference (*p* < 0.05). Mal: malaria, Db: diabetic, OCB: *Ocimum basilicum*, MET: metformin.

### 3.7. Effects of OCB on Inflammatory Cytokines

Serum tumor TNF‐*α* and IL‐6 concentrations are presented in Figures 9 and 10, respectively. No statistically significant (*p* > 0.05) difference was observed in TNF‐*α* concentrations among the experimental groups, indicating that neither the disease conditions nor OCB treatment produced a detectable alteration in this cytokine under the experimental conditions used. In contrast, IL‐6 exhibited a treatment‐responsive pattern. OCB‐treated groups showed significantly lower IL‐6 concentrations than the comorbid untreated mice, with the reduction reaching statistical significance at *p* < 0.01. The contrasting responses of TNF‐*α* and IL‐6 indicate that the inflammatory response was not uniformly altered across the measured cytokines. The reduction in IL‐6 in OCB‐treated animals, particularly in the comorbid condition, suggests a selective attenuation of this inflammatory marker following treatment.

**FIGURE 9:**
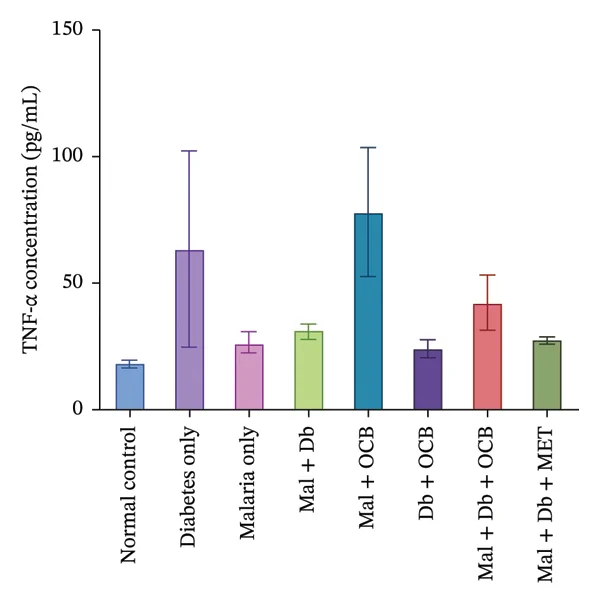
Effect of OCB on serum TNF‐*α* concentration in mice. Data are presented as mean ± SEM; *n* = 7. Different letters on bars indicate a significant difference (*p* < 0.05). Mal: malaria, Db: diabetic, OCB: *Ocimum basilicum*, MET: metformin.

**FIGURE 10:**
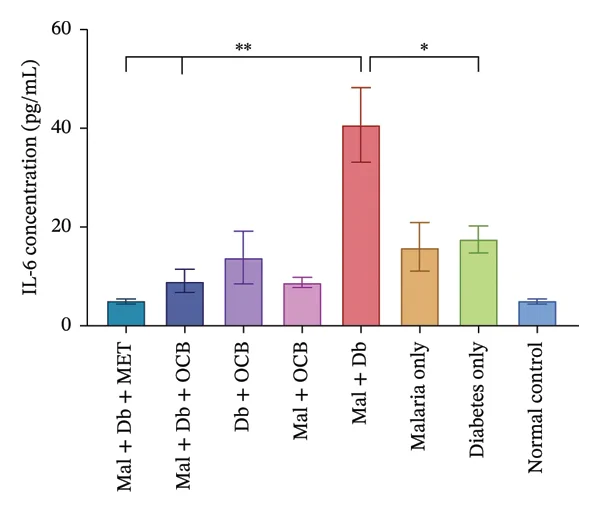
Effect of OCB on serum IL‐6 concentrations in mice. Data are presented as mean ± SEM; *n* = 7; ^∗^ *p* < 0.05, ^∗^^∗^ *p* < 0.01. Mal: malaria, Db: diabetic, OCB: *Ocimum basilicum*, MET: metformin.

### 3.8. Effects on PCV

PCV measured on Day 25 is presented in Figure 11. The Mal + Db group had a lower PCV than the corresponding OCB‐treated group, while PCV was significantly (*p* < 0.05) higher in both the Mal + Db + OCB and Mal + Db + Met groups than in untreated Mal + Db mice. The improvement in PCV following OCB treatment indicates attenuation of the reduction in PCV associated with the diabetes–malaria comorbid condition. This finding is particularly relevant in the context of malaria, where alterations in erythrocyte status may accompany increased parasite burden.

**FIGURE 11:**
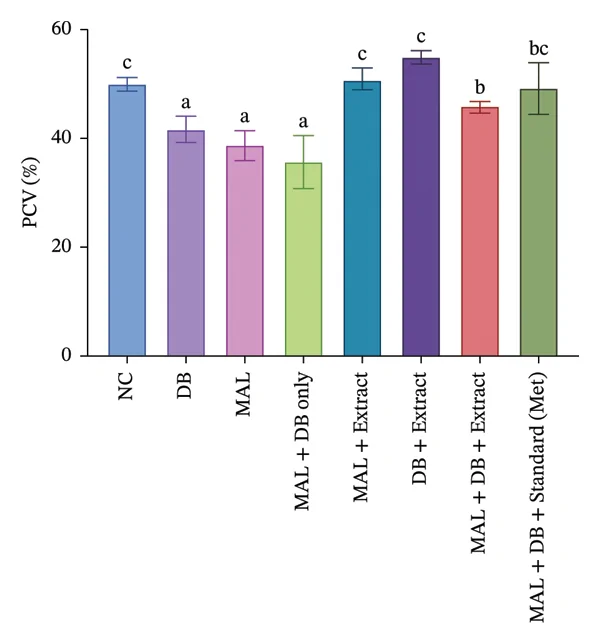
Packed cell volume (%) of experimental mice on Day 25. NC: normal control, MAL: malaria, DB: diabetic, OCB: *Ocimum basilicum*, Met: metformin.

### 3.9. Effects on Coagulation Parameters

The effects of OCB on aPTT and PT are presented in Figures 12 and 13, respectively. aPTT was significantly longer in the normal control group than in the experimental groups, indicating differences in the coagulation profile associated with the disease conditions. In malaria‐infected mice, a shorter but not significantly different aPTT was recorded with OCB treatment compared with the malaria‐only group. This may indicate a shift in the measured intrinsic/common coagulation response.

**FIGURE 12:**
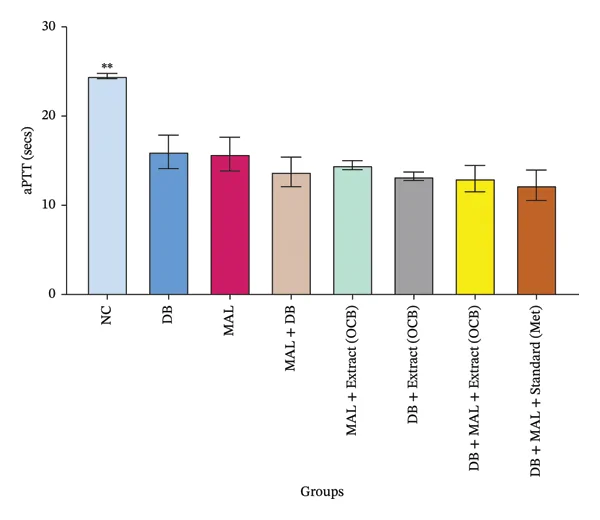
Activated partial thromboplastin time of experimental mice. Values represented as mean ± SEM, *n* = 7, ^∗^^∗^ *p* < 0.01, significantly higher than other groups. NC: normal control, MAL: malaria, DB: diabetic, OCB: *Ocimum basilicum*, MET: metformin.

**FIGURE 13:**
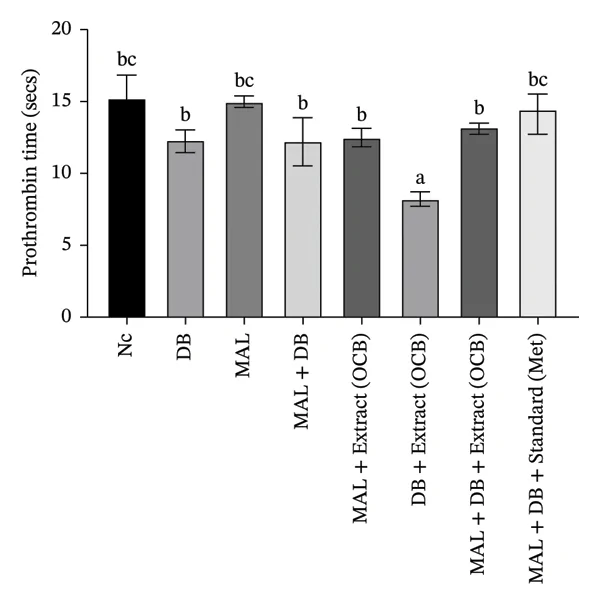
Prothrombin time of experimental mice. Values represented as mean ± SEM, *n* = 7. Different letters on bars indicate a significant difference (*p* < 0.05). NC: normal control, MAL: malaria, DB: diabetic, OCB: *Ocimum basilicum*, MET: metformin.

For PT, the diabetes‐only group showed a shorter clotting time than the normal control, establishing the reported procoagulant state in DM. OCB treatment did not significantly alter PT across most experimental groups, although a treatment‐related difference was observed in the Db + OCB group.

### 3.10. Plasma and Liver TP

The effects of OCB on plasma and liver TP concentrations are presented in Figure 14. Plasma TP was significantly reduced in the Mal + Db group compared with the normal control (Figure 14a), indicating an alteration in systemic protein status under the comorbid condition. OCB treatment produced a modest improvement in plasma TP relative to the untreated comorbid group, suggesting partial attenuation of this alteration.

**FIGURE 14:**
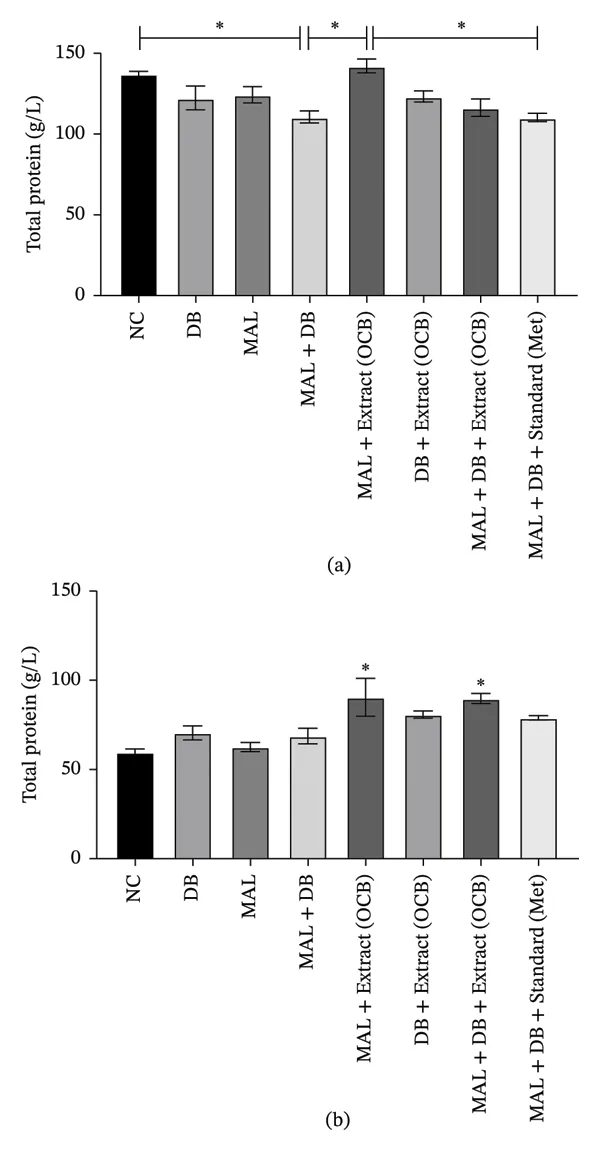
Plasma (a) and liver total protein (b) concentrations of experimental mice. Values are presented as mean ± SEM, *n* = 7, ^∗^ *p* < 0.0 significantly different. NC: normal control, MAL: malaria, DB: diabetic, OCB: *Ocimum basilicum*, MET: metformin.

In contrast, liver TP concentrations were higher in the malaria‐treated groups than in the normal control (Figure 14b). The difference between the plasma and hepatic TP responses indicates that systemic and hepatic protein profiles did not change in parallel following disease induction. The observed treatment‐associated changes therefore suggest that OCB may influence protein homeostasis differently at the circulating and hepatic levels.

## 4. Discussion

The present study investigated the effects of OCB leaf hydromethanol extract on parasitemia, metabolic, hepatic, renal, inflammatory, hematological, and coagulation outcomes in mice with diabetes–malaria comorbidity. Overall, OCB treatment was associated with attenuation of hyperglycemia and parasitemia, preservation of body weight and PCV, and improvement in several biochemical indices. The response was particularly evident in the comorbid condition, where OCB reduced FBG, parasitemia, ALT, AST, creatinine, and interleukin‐6 (IL‐6). These findings are relevant because diabetes and malaria can interact through metabolic dysregulation, inflammation, oxidative stress, and impaired host responses, potentially aggravating the clinical consequences of either condition [5].

The GC–MS analysis of OCB revealed a chemically diverse profile comprising 43 identified compounds, with fatty acids, phytosterols, flavonoid derivatives, terpenoid‐related compounds, hydrocarbons, phenolic compounds, and nitrogen‐ and sulfur‐containing compounds represented in the extract. The predominance of these compounds suggests that lipid‐derived constituents, phytosterols, and methoxylated flavonoids constitute important components of the extract. Previous studies have associated basil‐derived phytochemicals with antioxidant, anti‐inflammatory, antimicrobial, and other pharmacological activities [13–15].

The significant elevation of FBG in diabetic mice confirmed the establishment and persistence of the diabetic phenotype. The reduction in FBG following OCB administration in diabetic and malaria‐diabetic mice indicates that the extract retained glucose‐lowering activity even in the presence of malaria infection. This observation is consistent with experimental evidence demonstrating the antidiabetic potential of OCB [16, 17]. The glucose‐lowering effect observed in the present study may therefore reflect multiple complementary mechanisms, including reduced intestinal carbohydrate hydrolysis, enhanced pancreatic *β*‐cell activity and insulin sensitivity, improved glucose uptake in peripheral tissues, and inhibition of *α*‐glucosidase activity [18, 19].

The reduction in parasitemia following OCB treatment provides evidence of an antiplasmodial effect of the extract. The response became more apparent from Day 9 and was observed in both malaria‐only and malaria–diabetes groups, suggesting that the antiplasmodial activity of OCB was retained under the metabolically stressful comorbid condition. The finding is important because the interaction between diabetes and malaria may be bidirectional, with metabolic and immune disturbances potentially influencing parasite persistence and disease progression [5]. The observed antiplasmodial effect may be partly influenced by improved glycemic regulation, since restoration of metabolic homeostasis could contribute to improved host responses to infection. Interestingly, metformin treatment also produced a significant reduction in parasitemia in the diabetic‐malaria group at Day 25. Although metformin was included primarily as the standard antidiabetic comparator, this observation is consistent with experimental evidence that metformin can influence *Plasmodium* infection independently of its conventional glucose‐lowering role [20, 21]. In murine malaria models, metformin pretreatment has been reported to reduce parasitemia and to reverse infection‐associated suppression of hepatic AMPK activity and alterations in lipid metabolism [22]. One proposed explanation is that metformin‐induced AMPK activation suppresses anabolic and lipogenic processes in the host, potentially reducing the metabolic environment that supports parasite proliferation [21]. The reduction in parasitemia observed with metformin in the present study should be regarded as a potential ancillary antiplasmodial effect rather than evidence that metformin is an established antimalarial therapy. Importantly, the delayed reduction observed with metformin compared with the earlier decline apparent following OCB treatment suggests that the two interventions may not act through identical mechanisms. While metformin may influence parasite burden indirectly through host metabolic regulation, the response to OCB could reflect the combined actions of multiple plant‐derived constituents with direct and/or host‐mediated antiparasitic effects. Further study is required to establish the mechanisms responsible for these effects.

The attenuation of body weight loss in OCB‐treated mice further indicates an improvement in the overall physiological response to disease. Diabetes and malaria can both promote negative energy balance through altered glucose utilization, increased metabolic demand, reduced nutrient utilization, and systemic inflammatory responses [23, 24]. The more pronounced preservation of body weight in the diabetic and comorbid treated groups may therefore reflect improved metabolic control and reduced disease‐associated physiological stress. Flavonoid‐rich plant extracts, including OCB, are known to preserve muscle protein and fat stores during metabolic stress [25].

The increase in PCV following OCB treatment in comorbid mice is also noteworthy. Malaria is frequently associated with reductions in hematocrit and hemoglobin resulting from erythrocyte destruction, altered erythropoiesis, and other inflammatory and vascular processes [26]. The higher PCV in the treated group compared with the untreated comorbid group therefore suggests attenuation of the hematological disturbance associated with the combined disease state. The effect may be related, at least in part, to the lower parasite burden observed following OCB treatment, because reduced parasite‐associated erythrocyte destruction could contribute to preservation of circulating red‐cell mass.

The hepatic enzyme findings provide further evidence of a treatment‐associated improvement in biochemical alterations induced by diabetes and malaria. Elevated ALT in the untreated diabetic, malaria, and comorbid groups indicates disruption of hepatic biochemical homeostasis under these disease conditions, with the greatest AST alteration occurring in the comorbid group. The reduction of ALT and AST following OCB administration suggests attenuation of this disturbance. This interpretation is supported by recent experimental evidence showing that OCB extracts can exert antioxidant and protective effects in models of hepatic injury. In an experimental model of acetaminophen‐induced hepatotoxicity, basil extract reduced lipid peroxidation, enhanced antioxidant enzyme activity, and lowered circulating ALT and AST [27]. More recent characterization of the polyphenolic fraction of OCB has likewise demonstrated antioxidant and protective activities, supporting the biological plausibility of these effects [28, 29].

The reduction in urea and creatinine concentrations suggests that OCB positively modified the renal biochemical response associated with the disease conditions. The reduction in urea in malaria‐infected mice may be particularly relevant because malaria‐associated systemic illness can contribute to altered renal function through dehydration, hemolysis, inflammation, and other mechanisms. Previous experiments also support a renal protective potential of OCB [30]. Furthermore, the lower creatinine concentrations observed in the OCB‐treated groups, including values below the normal control level, indicate modulation of renal biochemical markers.

The inflammatory findings suggest that OCB may selectively modulate the inflammatory response. Although TNF‐*α* did not differ significantly among the experimental groups, IL‐6 was reduced following OCB treatment, particularly in the comorbid condition. The absence of a significant TNF‐*α* response alongside the reduction in IL‐6 indicates that the inflammatory effects of OCB were not uniform across cytokines. This distinction matters because diabetes and malaria involve complex, dynamic inflammatory responses rather than a simple elevation of all inflammatory mediators. Recent literature on OCB describes antioxidant, anti‐inflammatory, and immunomodulatory activities associated with its polyphenolic and volatile constituents, including modulation of inflammatory signaling pathways and cytokine production [13]. The reduction in IL‐6 observed here may therefore represent attenuation of part of the inflammatory response associated with diabetes–malaria comorbidity.

The coagulation findings showed that OCB treatment shortened aPTT in malaria‐infected mice relative to the untreated malaria group; however, this change did not normalize toward the normal control, which exhibited a significantly longer aPTT than all experimental groups. Rather, the OCB‐associated reduction indicates a further shift toward a shorter clotting time within the intrinsic/common coagulation pathway. Although a shortened aPTT may be compatible with faster clot formation, it cannot, in isolation, be interpreted as evidence of increased thrombotic risk or a generalized procoagulant effect. This distinction is particularly important in malaria, in which hemostatic disturbances may involve simultaneous activation of coagulation and consumption of coagulation components, with the severity and direction of these changes varying according to disease status [31, 32]. The largely unchanged PT following OCB treatment further suggests that the effect was not uniform across the measured coagulation pathways. Therefore, the present findings indicate that OCB modulated the coagulation response differently depending on the pathological context, but the biological significance of the aPTT shortening remains uncertain. Additional assessment of other key coagulation parameters such as fibrinogen, D‐dimer, and thrombin‐generation would be required to determine whether this alteration has implications for hemostatic or thrombotic risk.

The changes in TP demonstrate that diabetes–malaria comorbidity altered systemic protein homeostasis. The significant reduction in plasma TP in the comorbid group may reflect the combined effects of metabolic disturbance, infection‐associated catabolism, altered protein synthesis, and changes in plasma protein distribution. OCB produced only a relatively slight improvement in plasma TP, indicating that its effects on protein homeostasis were less pronounced than its effects on glucose, parasite burden, or selected organ function markers.

Collectively, the findings indicate that OCB exerted multidimensional effects in the diabetes–malaria model, with the most consistent responses involving glycemic control, parasite burden, body weight preservation, selected hepatic and renal biochemical markers, IL‐6, and PCV. The simultaneous improvement in metabolic and infection‐related outcomes is particularly relevant because diabetes and malaria can interact through overlapping inflammatory, oxidative, metabolic, and immune pathways [5]. The study therefore provides experimental evidence supporting further investigation of OCB as a candidate plant‐derived intervention for diabetes–malaria comorbidity.

## 5. Conclusion

The present study demonstrates that OCB methanol extract produced beneficial effects across several metabolic, parasitological, biochemical, inflammatory, hematological, and coagulation outcomes in mice with diabetes–malaria comorbidity. OCB treatment reduced FBG and parasitemia, attenuated disease‐associated body weight loss, and improved PCV in comorbid mice. The treatment was also associated with favorable changes in selected hepatic and renal biochemical markers and a significant reduction in IL‐6, suggesting attenuation of metabolic, inflammatory, and organ‐related disturbances associated with the combined disease state.

The reduction in parasitemia together with improved glycemic control is particularly noteworthy, as these effects occurred in the presence of diabetes–malaria comorbidity, where metabolic and infectious stress may interact to aggravate disease outcomes. The observed modulation of aPTT and limited effect on PT further suggests that OCB may influence hemostatic responses, although the precise mechanisms and clinical significance of these coagulation effects require further investigation.

Overall, the findings provide preclinical evidence supporting the potential of OCB as a promising phytopharmacological source for treating and managing diabetes–malaria comorbidity. Further studies incorporating parasite‐specific mechanistic assays and glucose and insulin homeostasis are warranted to establish the mechanisms underlying the observed effects.

## Author Contributions

Conceptualization: Ayodele Opeyemi Oluwayemisi and Oyerinde Olawunmi Rashidat; investigation: Ayodele Opeyemi Oluwayemisi, Oyerinde Olawunmi Rashidat, Bello Oluwatosin Nancy, and Adeigbe Daniel Gbolahan; data curation and analysis: Ayodele Opeyemi Oluwayemisi, Oyerinde Olawunmi Rashidat, Bello Oluwatosin Nancy, Adeigbe Daniel Gbolahan, and Adegbite Gbenga; writing of original draft: Ayodele Opeyemi Oluwayemisi and Oyerinde Olawunmi Rashidat; manuscript review and editing: Ayodele Opeyemi Oluwayemisi and Adegbite Gbenga; visualization: Femi‐Olabisi Fehintoluwa Joy; and supervision: Ayodele Opeyemi Oluwayemisi and Oyerinde Olawunmi Rashidat.

## Funding

The author(s) received no financial support for the research.

## Disclosure

All authors have read and agreed to the published version of the manuscript. A preprint version of this manuscript has been previously deposited on bioRxiv (doi: https://doi.org/10.1101/2025.09.13.676048) [33].

## Conflicts of Interest

The authors declare no conflicts of interest.

## Data Availability

The data that support the findings of this study are available from the corresponding author upon reasonable request.
